# Pharyngotonsillitis in children: view from a sample of pediatricians and otorhinolaryngologists

**DOI:** 10.1016/S1808-8694(15)30845-4

**Published:** 2015-10-18

**Authors:** Aracy Pereira Silveira Balbani, Jair Cortez Montovani, Lidia Raquel de Carvalho

**Affiliations:** 1 PhD. Volunteer professor of Otorhinolaryngology and Head and Neck Surgery - Botucatú Medical School (UNESP).; 2 Associate Professor of Otorhinolaryngology and Head and Neck Surgery - Botucatú Medical School (UNESP).; 3 Assistant Professor - Department of Biostatistics - Instituto de Biociências da UNESP. Otorhinolaryngology and Head and Neck Surgery Program - Botucatú Medical School - Faculdade de Medicina de Botucatu (UNESP).

**Keywords:** peritonsillar abscess, child, rheumatic fever, streptococcus pyogenes, tonsillitis

## Abstract

Acute pharyngotonsillitis is a common upper airway infection in children. **Aim:** To analyze opinions and practices of pediatricians and otorhinolaryngologists from Sao Paulo State, Brazil, concerning diagnosis, treatment and prevention of pharyngotonsillitis and their complications in children. **Methods:** We randomly selected 1,370 pediatricians and 1,000 otolaryngologists from Sao Paulo State, Brazil. A questionnaire was mailed to the specialists. **Study design:** Cross-sectional. **Results:** 95.8% of the pediatricians and 91.5% of the otolaryngologists do not perform routine laboratory diagnosis for acute pharyngotonsillitis in children. The antimicrobials more commonly prescribed by pediatricians for treatment of bacterial pharyngotonsillitis were: oral penicillin for 10 days (33.6%) and s single injection of benzathine penicillin G (19.7%). The antimicrobials prescribed more often by otorhinolaryngologists for treatment were: oral penicillin for 10 days (35.4%) and oral penicillin for 7 days (25.7%). Tonsillectomy was considered the most effective measure for prevention of bacterial pharyngotonsillitis by more than half of pediatricians and otolaryngologists. Repeated pharyngotonsillitis was the main reason for otolaryngologists to indicate tonsillectomy for school-aged children and adolescents (49.3% and 53.4% respectively). **Conclusions:** It is necessary to standardize the practices of pediatricians and otolaryngologists regarding diagnosis and treatment of pharyngotonsillitis in children.

## INTRODUCTION

Acute pharyngotonsillitis are frequent upper airway infections in children and teenagers. Most cases, especially in infants, is of viral etiology (rhinovirus, Epstein-Barr virus, parainfluenza and influenza) 1, 2.

Among bacterial pharyngotonsillitis we stress those caused by Streptococcus pyogenes, Lancefield Group A Beta-Hemolytic Streptococcus, which can cause suppurative complications (cervical adenitis; peritonsillar, retropharyngeal or neck abscesses) and non-suppurative (rheumatic fever, acute diffuse glomerulonephritis, and auto-immune neuropsychiatric disorders - PANDAS)^3^.

According to Santos; Berezin (2005)1, clinical signs and symptoms belonging to pharyngotonsillitis are enough to differentiate viral from bacterial etiology. To assess 376 children from two to 13 years of age with acute pharyngotonsillitis seen at the pediatric emergency ward, the authors observed that palate petechiae, palatine tonsil effusion and the painful neck nodes were significantly more common in children with positive culture for S. pyogenes (24.4% of the total); however, these signs had a low positive predictive value (31-49%). Based only on the clinical exam, pediatricians indicated antibiotic treatment for 47% of the children who had negative oropharyngeal secretion cultures. On the other hand, the physician did not identify the bacterial etiology in 21% of the children with positive culture. These results indicate the usefulness of a microbiological diagnosis in children with acute pharyngotonsillitis.

The gold standard in the diagnosis of streptococcal pharyngotonsillitis is the culture of pharyngeal tonsils secretion and also that from the posterior oropharyngeal wall in blood agar dish with goat blood at 5%^2^, ^3^. This test has over 90% sensitivity, however the result takes 24 to 48 hours to be ready^4^.

One diagnostic alternative is the quick antigenic test. The kits for this test take S. pyogenes antigens from oropharyngeal swabs, providing the results in a few minutes. This method is highly sensitive (80-95%) and specific (90%)^3^.

Anti-streptolysin O (ASLO) values usually go up one week after the streptococcal infection reach its peak in three to six weeks, then fall back^3^. According to Machado et al. (2001)^5^ ASLO’s normal ranges vary according with the individual’s age, the season of the year, geographic location and prevalence of streptococcal infections in the population. Infants and pre-school-aged children have lower ASLO values when compared with school-aged children because of their lower exposure to streptococcal antigens.

S. pyogenes is considered universally sensitive to penicillin. The first choice antimicrobial agents are^4^: phenoxymethylpenicillin (Oral V Penicillin V) or 10 days of amoxicillin, or even a single intramuscular injection of G-Benzathine Penicillin.

Failure in the treatment with V penicillin can happen in up to 35% of the cases, especially in children younger than six years. According to Cohen (2004)^6^ reasons for treatment failure are: incorrect dosage use or drug use for less than 10 days, new child contact with the individual infected by S. pyogenes, penicillin degrading by the beta-lactamases produced by the oropharyngeal flora, or eradication of the oropharyngeal protecting flora (Streptococcus salivarius and other alpha-hemolytic streptococci). When one suspects of penicillin degrading, beta-lactamase inhibitors must be used (amoxicillin/clavulanate, or amoxicillin and sulbactam).

For patients with penicillin allergy, we can use erythromycin for 10 days, or azithromycin for 5 days^6^. S. pyogenes strains are quick to develop macrolide resistance, and the literature does not recommend azithromycin as first treatment option^4^. Sulfonamides and tetracyclines are not indicated to treat streptococcal pharyngotonsillitis because of the high rate of failure in bacterial eradication^2^, ^3^.

Thanks to antibiotic treatment, fewer children are being submitted to tonsillectomy surgery because of repetitive bacterial pharyngotonsillitis. Surgery is indicated when the child has seven infectious episodes in one year, five episodes per year in two years or 3 episodes per year in three consecutive years^7^.

Rheumatic fever is the main cause of chronic acquired cardiopathy in children and adolescents in our country^8^. The disease diagnosis is performed according to Jones’s criteria, established by the American Heart Association and revised in 1992. The presence of two major criteria or one major and two minor, associated with proof of previous streptococcal infection is highly suggestive of rheumatic fever (Table 1). Chorea is the only criterion that, alone, is diagnostic of the disease^8^. High titers of ASLO only indicate a recent infection by S. pyogenes, however, alone, it does not establish the diagnosis of acute rheumatic fever, nor measure disease activity^5^.

**Table 1:** Diagnostic criteria for rheumatic fever.

Major signs	Carditis
	arthritis
	chorea
	subcutaneous nodules
	marginal Erythema
Minor signs	Fever
	arthralgia
	VHS elevation and/or PCR* and/or a-1 acid glycoprotein
	PR space lengthening in the electrocardiogram
Evidence of previous streptococcal infection	Positive oropharyngeal culture for S. pyogenes
	High titles of anti-streptococcal antibodies (ASLO, anti-hyaluronidase, anti-DNAse B, anti-streptokinase)

* ESR: Erythrocyte sedimentation rate; CRP: C-Reactive Protein

The goal of the present investigation was to study the opinions and treatment approaches of pediatricians, and otorhinolaryngologists from the State of São Paulo in terms of diagnosis, treatment and prevention of pharyngotonsillitis and its complications in children.

## METHODS

From the Society of Pediatrics and Otorhinolaryngology of São Paulo State we randomly selected 1,370 pediatricians and 1,000 otorhinolaryngologists from São Paulo. From August to November of 2006, we mailed to these specialists a form with the following issues:

a) Professional profile: gender; year of graduation; academic title; place of work (São Paulo City or town in the country/coast); type of activity (in private clinic, public health care institution, university hospital, day-care, orphanage, etc.).
b) “As you see a previously healthy child with acute symptoms of sore throat and fever, and during the physical exam you notice hyperemia and exudate in the palatine tonsils, do you routinely order some laboratorial exam for diagnosis before starting treatment? (it is possible to have more than one answer)”.
  Answer alternatives were: does not order tests; orders CBC; orders oropharyngeal secretion culture and antibiogram; orders the quick strep test to detect Streptococcus pyogenes on the oropharynx (please state whether you do the test in your office or if you refer the patient to a lab); order anti-streptolysin O (ASLO) levels, or other tests (please specify).
c) Your opinion about the quick strep test to detect Streptococcus pyogenes in the diagnosis of cases of acute pharyngotonsillitis in children in your daily practice. Possible answers: the test is useful in all cases; useful only in cases when there is no visible purulent effusion in the tonsils; useful only in institutionalized children (day-care and orphanages); or you do not use the test because it is not available in the region where you practice.
d) “Let’s say you see a child of pre-school age with sore throat and fever (38.5°C) for 24 hours now, the mother medicated the child with analgesic and antifever drugs. During physical exam, the child has hyperemia and purulent effusion in the palatine tonsils. The child is not allergic to any drug and is able to swallow food despite the sore throat. Since this is a case of bacterial pharyngotonsillitis, which is your antibacterial drug of choice (agent and treatment duration) “?
  Answer alternatives were: single injection of benzathine penicillin; injectable lincomycin; injectable third generation cephalosporin (Ceftriaxone); oral penicillin (Ampicillin, amoxicillin); penicillin with beta-lactamase inhibitor (augmented amoxicillin with potassium clavulanate, Ampicillin sulbactam); first, second or third generation oral cephalosporin; sulfa and associations (sulfamethoxazole + trimethoprim); azithromycin; other macrolides (erythromycin, clarithromycin) or another antimicrobial agent, please state the number of doses or treatment time for the treatment option chosen.
e) “In the last 12 months, did you see any child with peritonsillar abscess or cervical adenitis as a suppurative complication of bacterial pharyngotonsillitis?” (yes or no, please state how many cases).
f) In the last 12 months, did you see any child with rheumatic disease as a non-suppurative complication of streptococcal pharyngotonsillitis? (yes or no and please state the number of cases).
g) Knowledge about the criteria used to diagnose rheumatic fever in children (excellent, good, regular or non-satisfactory).
h) Opinion about the following to prevent repetition bacterial pharyngotonsillitis in children: hygiene education (e.g. not to share plates and silverware with other people); homeopathy; vitamin supplements; thymomodulin (Leucogen®); Bacterial lysate agents (Biostim®, Broncho-Vaxom®, Estimoral®, etc.); Pelargonium sidoides (Kaloba®, Umckan®); tonsillectomy and treatment of asymptomatic patients with Streptococcus pyogenes who live with the child. Each option was classified as: very efficient, little efficient or non-efficient.
i) Familiarity with the results of recent studies aimed at developing a vaccine against Group A Beta-Hemolytic Streptococcus (Streptococcus pyogenes) divulged in the scientific literature (excellent, good, regular or non-satisfactory).
j) To the pediatricians we asked: “Do you consider necessary to operate a child (tonsillectomy) who has already had one episode of peritonsillar abscess or cervical adenitis as suppurative complications of bacterial pharyngotonsillitis?” (yes or no?).
k) Otorhinolaryngologists were also asked about the most frequent reason to indicate a tonsillectomy (repetition bacterial pharyngotonsillitis, upper airway obstruction or chronic tonsillitis with caseous/halitosis), according to the patients’ age (pre-school, school age, teenagers and adults).
l) Comments on the diagnosis and treatment of pharyngotonsillitis in children (open answer question).

The participants had the option to identify themselves or not in the answer sheet.

The data was submitted to statistical analysis by the chi-squared test through the SAS software, version 6.12, with 5% as significance level.

## RESULTS

294 filled questionnaires were returned to us from pediatricians (21.4% of the total mailed) and 144 from otorhinolaryngologists (14.4%).

a) Professional profiles

**Graph 1:**
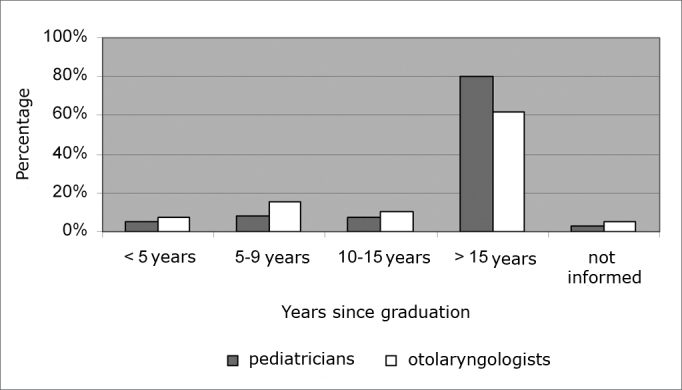
Time after graduation of the study participants.

188 pediatricians (64%) and 49 ENTs (34.1%) were females; 106 pediatricians (36%) and 95 ENTs (65.9%) were males. Most of the pediatricians and ENTs who participated in the study (80% and 61.8% respectively, p<0.0001) had more than 15 years of experience in their fields (Graph 1). As to academic titles, there was a predominance of professionals who concluded a medical residency: 248 pediatricians (84.4%) and 96 ENTs (66.7%); p<0.0001.

233 pediatricians (75.8%) and 105 ENTs (72.9%) worked in the country; 67 pediatricians (22.8%) and 33 ENTs (22.9%) were from São Paulo City; p<0.0001. Most of the professionals worked in the public health care system and in the private system (69.8% of the pediatricians and 61.1% of the ENTs), there were 48 pediatricians (16.3%) and 45 ENTs (31.2%) associated with University Hospitals.

Time after graduation of the study participants.

b) Approach to diagnose a fictitious case of acute pharyngotonsillitis in children

275 pediatricians (95.8%) and 130 ENTs (91.5%) answered they would not routinely order lab tests to diagnose acute pharyngotonsillitis in children (p<0.0001). CBC would be ordered by 2.1% of the pediatricians and 5.6% of the ENTs.

Three ENTs (2%) would order the quick strep test to detect Streptococcus pyogenes in the oropharyngeal secretion, two would refer the patient to a lab for secretion collection, and one of them would do it in his own office. Two ENTs (1.3%) would order a CBC and ASLO levels and oropharyngeal secretion culture and antibiogram.

Only two pediatricians (0.6%) would order the quick strep test, harvested in a lab. One specialist would order the quick test and the CBC; one would order CBC and ASLO levels; and one would order only the oropharyngeal secretion culture and antibiogram.

c) Opinion on the quick antigenic test (strep test) to detect Streptococcus pyogenes in the oropharyngeal secretion.

Most of the pediatricians (76.9%) and ENTs (69%) do not use this diagnostic method because it is not available where they practice (p<0.0001).

Among the pediatricians, 28 (9.6%) consider the test useful for diagnosis in all cases; 20 (6.9%) deem it useful in cases when there is not visible effusion on the palatine tonsils and 6 (2.1%) deem it useful for diagnosis of institutionalized children (in day-care centers or orphanages).

**Graph 2:**
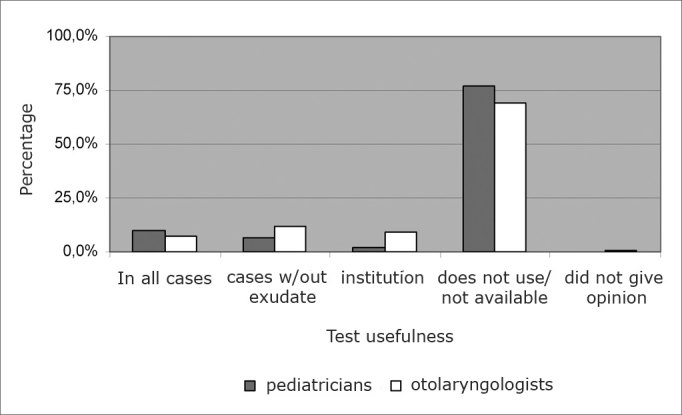
Opinion of pediatricians and ENTS about the usefulness of the quick antigenic test to detect S. pyogenes in the diagnosis of acute pharyngotonsillitis in children.

Among ENTs, 10 (7%) deem the test useful for diagnosis in call cases; 17 (11.9%) deem it useful in cases when there is no visible effusion in the palatine tonsils and 13 (9.1%) deem it useful to diagnose institutionalized children (Graph 2).

d) Antibiotic agent options to treat acute bacterial pharyngotonsillitis in pre-school aged children.

In this issue, pediatricians and ENTS had 41 and 32 different answers, respectively.

The treatment schemes preferred by pediatricians were: oral penicillin for 10 days (33.6%, p<0.0001), single dose benzathine penicillin (19.7%), and oral penicillin for 7 days (9.2%). Nine specialists (3%) recommended the use of beta-lactamase inhibitor for 10 days.

Among ENTs, 51 (35.4%) prescribed oral penicillin for 10 days and 37 (25.7%) prescribed it for 7 days. Fourteen (9.7%) recommended the use of penicillin with a beta-lactamase inhibitor for 10 days, and four (2.8%) during seven days. Only four ENTs (2.8%) chose benzathine penicillin in a single dose.

Azithromycin was the treatment of choice of 7.1% of the pediatricians and 4.1% of the ENTs, and they preferred the use of three doses. Treatment with first or second generation cephalosporins for seven days was mentioned, each one, by three ENTs (2%). Injectable lincomycin was recommended in a single dose by one pediatrician and in five doses by one ENT.

e) cases of peritonsillar abscess or neck adenitis

In the 12 months before this study, these complications had been seen by 109 pediatricians (37%), at a frequency that varied from one to 12 cases and by 65 ENTs (45.2%), also in a varied number (one to 20 cases).

f) cases of rheumatic disease as a complication of streptococcal pharyngotonsillitis in children

In the 12 months before the study, 64 pediatricians (21.7%, p<0.0001) and 27 ENTs (18.7%, p<0.0001) had five pediatric patients with rheumatic fever, being between one and five cases. One pediatrician and one ENT did not answer this question.

g) familiarity regarding the criteria used to diagnose rheumatic fever

**Graph 3:**
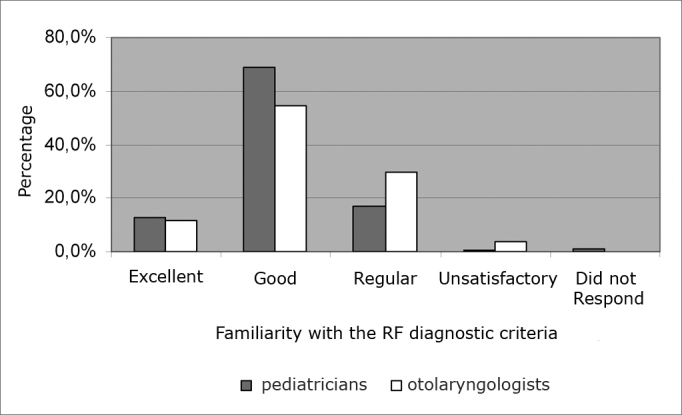
ENTs’ and pediatricians familiarity regarding the diagnostic criteria of rheumatic fever (FR).

202 pediatricians (68.7%, p<0.0001) and 79 ENTs (54.8%, p<0.0001) deemed good their familiarity with rheumatic fever diagnostic criteria (Graph 3). Three pediatricians did not answer this question.

h) Efficacy of repetition bacterial pharyngotonsillitis prevention in children

The most efficacious preventive measures as seen by the pediatricians were: tonsillectomy (55.8%) and treatment of the S. pyogenes asymptomatic carriers who lived with the children (42.8%). The so considered ineffective measures were: use of vitamins (48.6%), Pelargonium sidoides stratum (45.2%), thymomodulin (43.2%) and homeopathy (41.8%).

The most efficient preventive measured as seen by ENTs was tonsillectomy (84%, p<0.0001); and the least efficacious was the use of vitamins (40.3%) (Table 2).

**Table 2:** Pediatricians’ and ENTs’ opinions about the efficacy of bacterial pharyngotonsillitis prevention measures in children.

	Very efficient (%)		Little efficient (%)		Unneficient (%)		Did not give opinion (%)	
	Ped	ENT	Ped	ENT	Ped	ENT	Ped	ENT
Hygiene	35,7	16,6	40,1	46,5	20,4	32,6	3,8	4,3
Homeopathy	10,5	4,8	38,8	55,5	41,8	31,9	8,9	7,8
Vitamins	5,4	10,4	40,6	45,1	48,6	40,3	5,4	4,2
Thymomodulin	15	13,9	34,3	63,9	43,2	15,3	7,5	6,9
Bacterial remains	11,3	27,8	38,4	59	38,1	9	12,2	4,2
*P. sidoides*	3	8,3	34,3	44,4	45,2	34,7	17,5	12,6
Tonsillectomy	55,8	84	24,4	7,6	10,9	2,7	8,9	5,7
Healthy carrier treatment	42,8	20,1	37,9	39	12,9	27	6,4	13,9

i) familiarity with the study regarding the development of a vaccine against S. pyogenes

**Graph 4:**
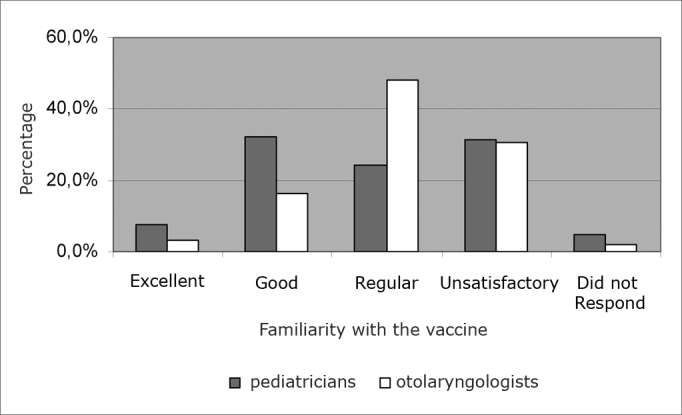
Pediatricians’ and ENT’s familiarity with studies regarding the vaccine against *S. pyogenes*.

141 pediatricians (48%) and 46 ENTs (32%) deemed as good their knowledge on the development of a vaccine against S. pyogenes. On the other hand, 30.6% of the pediatricians and 31.2% of the ENTs deemed it not satisfactory (Graph 4).

j) Pediatricians’ opinions about tonsillectomy surgery in the patient who has already presented one episode of peritonsillar abscess or a neck adenitis.

The tonsillectomy surgery in children who had a suppurative complication of bacterial pharyngotonsillitis was considered not necessary by 188 pediatricians (64.1%, p<0.0001) and necessary by 87 (29.6%). Eighteen specialists did not answer.

k) ENTs’ indication for tonsillectomy in the different age ranges

**Graph 5:**
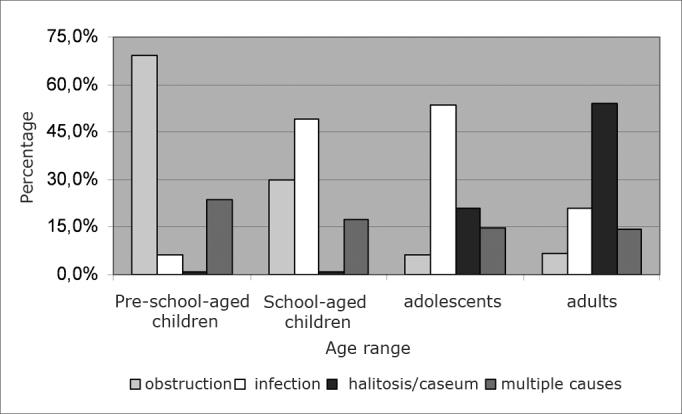
Reasons for indicating tonsillectomy surgery by ENTs in the different age ranges.

The major indication for tonsillectomy in pre-school children was upper airway obstruction (69.4%, p<0.0001); in school-aged children and in adolescents, repetition pharyngotonsillitis (49.3% and 53.4%, respectively), and in adults, tonsillitis with halitosis and caseum (54.1%) (Graph 5). Seven specialists did not answer the question.

l) Comments on the diagnosis and treatment of pharyngotonsillitis in children.

Two ENTs stated that the diagnosis of pharyngotonsillitis is clinical; however, the specialist “rarely palpates the patient’s abdomen”, and thus does not see the liver and spleen enlargement in cases of infectious mononucleosis. Seven specialists also deemed it necessary to investigate other diseases in children with repetition pharyngotonsillitis: oral breathing, atopy, gastro-esophageal reflux and malnourishment.

Still, regarding diagnosis, 3% of the pediatricians and 4% of the ENTs complained of the high cost of the quick strep test kit used for the antigenic detection of S. pyogenes, which makes it difficult to carry out such test in the clinical practice, especially in the public health-care system.

The overprescription of antibiotics to treat sore throats in children was the most frequent comment among pediatricians (10.2%) and ENTs (7%). Two ENTs remarked that “many patients already come to the office taking some antibiotic that was wrongly prescribed to them at the emergency ward”, and one mentioned self-medication. Among pediatricians, one remembered the laymen’s prescription of antimicrobial agents that happens at drug stores, and two discussed the overprescription of antibiotics by physicians for the following reason: “it is impossible to ask the child to return to the office for a reassessment in busy public health care facilities”, the “parents’ pressure” or even the “influence of the pharmaceutical industry advertisement “.

Thirty six pediatricians (12.2%) justified they choose a single dose benzathine penicillin and 10 day oral penicillin depending on: a) the patient’s social and economical level (“penicillin is an inexpensive drug and usually available in the public health care system”) or b) the issue of drug administration practice. Five pediatricians stated that “many hospitals refuse to administer benzathine penicillin when the prescription comes from a physician that is not a member of its clinical staff”. Three physicians stated that “there are parents who refuse to have their children take such injection”; one said he prescribed benzathine penicillin “only when the child is vomiting”, and another colleague only prescribes it for children above 4 years of age “.

Two pediatricians stated that they do not prescribe antimicrobial drugs to treat pharyngotonsillitis in children below 3 years of age. It was also mentioned the need to educate the parents as to not discontinue the antibiotic treatment prescribed by the physicians.

In order to prevent repetition pharyngotonsillitis, a pediatrician prescribed levamisole as an immune stimulant, and there is one who tells the parents to stop taking their child to day care. One ENT prescribes iodine.

## DISCUSSION

As we propose a fictitious case of acute pharyngotonsillitis in children, we noticed that 95.8% of the pediatricians and 91.5% of the ENTs who participated in this study do not order tests for a laboratorial diagnosis. When they do it, usually they chose a complete blood count.

As a point well made by a pediatrician, “S. pyogenes is not the only pathogen involved in pharyngotonsillitis”9. However, this is an important bacteria to consider in cases of pharyngotonsillitis because of its potential to cause severe complications, such as rheumatic fever.

Most of the physicians in this sample stated that they do not use the quick antigenic test (quick strep throat test) to detect S. pyogenes because the method is not available where they practice. This was the most frequent answer, even among the professionals who worked in the City of São Paulo. Moreover, 16.3% of the pediatricians and 31.2% of the ENTs interviewed worked in a university setting, where one would expect the test should be part of routine urgent care of both specialties.

Many colleagues stressed the high cost of the quick antigen test kit, and this makes it difficult to use if routinely. It is desirable that in the future we should have less expensive commercial kits available, providing of its use in a greater number of places. This shall contribute to a better microbiological diagnosis of pharyngotonsillitis, avoiding the unnecessary use of antimicrobial agents in patients with viral infections.

There was a large variation in answers to the question about antimicrobial use in acute pharyngotonsillitis. Oral penicillin for 10 days was the preferred treatment by pediatricians and ENTs (33.6% and 35.4%, respectively), but we have observed some differences in the approaches used by the two groups of specialists. Pediatricians use more benzathine penicillin than ENTs (19.7% x 2.8%), while the latter prescribe penicillin with beta-lactamase inhibitor with greater frequency (9.7% x 3%). Although 10 days of antibiotic treatment is widely accepted to eradicate S. pyogenes from the oropharynx, 9.2% of the pediatricians and 35.4% of the ENTs prescribe treatment with penicillin for 7 days only.

The significant majority of professionals from these two specialties had not see children with cervical adenitis, peritonsillar abscess or rheumatic fever as a complication of bacterial pharyngotonsillitis in the 12 months prior to the study. On the other hand, one ENT from São Paulo City and another one from the country stated they had had 20 patients of peritonsillar abscess or neck adenitis during such time, which is a very concerning piece of data.

Most pediatricians and ENTs (68.7% and 54.8%, respectively) deemed they were well informed about the criteria used to diagnose rheumatic fever, an important fact in the identification of patients with such disease.

The only prevention measure for bacterial pharyngotonsillitis deemed efficient for more than half the pediatricians and ENTs was the tonsillectomy surgery. Homeopathy, the use of vitamins and thymomodulin were considered very little efficient by most of the ENTs and totally inefficient by the pediatricians.

The phytodrug Pelargonium sidoides was included in this study because the manufacturer recommends its use for some days after the end of UAWI (upper airway infections) symptoms disappear, as a “subsequent treatment, especially in the case of a chronic course of the disease or frequent recurrence “, in order to “avoid recurrences”. Treatment duration should not exceed three weeks^10^.

Approximately one third of the pediatricians and ENTs deemed they were not satisfactorily aware of the studies being carried out in order to develop a vaccine against the Group A beta-hemolytic streptococcus, pointing towards the need for a better scientific disclosure of these studies for both these specialties. One pediatrician mentioned that the vaccine against S. pyogenes is taking too long to be launched in the market.

ENTs pointed out that repetition pharyngotonsillitis is the major indication for tonsillectomy surgeries in school-aged children and adolescents. One specialist mentioned that, in the future, vaccination against S. pyogenes will reduce the number of patients submitted to surgery.

Among pediatricians, there were different points of view regarding tonsillectomy. One professional stated that he only considers the procedure necessary when the ASLO level exceeds 400 UI/ml, and another one believes that the extraction of the palatine tonsils is an “incorrect approach”. Most of them (64.1%) believe it is not necessary to perform tonsillectomies in children who have one episode of suppurative complication of pharyngotonsillitis. In fact, this surgical indication is controversial. According to Discolo (2003)3, the recurrence of peritonsillar abscesses during new episodes of pharyngotonsillitis in children who are not submitted to surgery vary between 7 and 17%.

To conclude, Table 3 summarizes the view points and approaches of pediatricians and ENTs in relation to pharyngotonsillitis in children.

**Table 3:** Summary of the opinions and treatment approaches of 294 pediatricians and 144 otorhinolaryngologists from the State of São Paulo about pharyngotonsillitis in children.

**A sample of pediatricians, ENTs and pharyngotonsillitis in children**
95.8% of the pediatricians and 91.5% of the ENTs do not order tests for laboratorial diagnosis.
The antimicrobial agents more frequently prescribed by pediatricians to treat acute pharyngotonsillitis were: oral penicillin for 10 days (33.6%) and a single dose of benzathine penicillin (19.7%).
The antimicrobial agents more frequently prescribed by ENTs to treat acute pharyngotonsillitis were: oral penicillin for 10 days (35.4%) and oral penicillin for 7 days (25.7%).
62.5% of the pediatricians and 54.8% of the ENTs did not have cases of peritonsillar adenitis or neck adenitis as a complication of pharyngotonsillitis in the 12 months that preceded the study.
97.9% of the pediatricians and 80.5% of the ENTs did not have cases of rheumatic fever in the 12 months that preceded the study.
68.7% of the pediatricians and 54.8% of the ENTs consider they have good familiarity with rheumatic fever diagnostic criteria.
48% of the pediatricians and 32% of the ENTs deemed they had good familiarity with the studies being conducted to develop a vaccine against S. pyogenes.
The prevention measure for bacterial pharyngotonsillitis deemed very efficient by more than half of the pediatricians and ENTs was tonsillectomy.
Repetition pharyngotonsillitis was the main reason for ENTs to indicate tonsillectomy surgery in school-aged children and adolescents (49.3% and 53.4%, respectively).

## CONCLUSIONS

Based on the results found in this paper, we suggest that continuing education programs for pediatricians and otorhinolaryngologists broaden their disclosure of scientific studies on the diagnosis and prevention of pharyngotonsillitis and, try to bring a common ground regarding treatment approach in both specialties.
